# Intensity-Modulated Radiotherapy Associated With Improved Survival Outcome in Anal Cancer

**DOI:** 10.3389/fonc.2022.911925

**Published:** 2022-05-26

**Authors:** Ahmed Allam Mohamed, Marsha Schlenter, Alexander Heinzel, Svetlana Kintsler, Michael J. Eble

**Affiliations:** ^1^ Department of Radiation Oncology, RWTH Aachen University, Aachen, Germany; ^2^ Deprtment of Nuclear Medicine, RWTH Aachen University, Aachen, Germany; ^3^ Department of Pathology, RWTH Aachen University, Aachen, Germany

**Keywords:** squamous cell carcinoma (SCC), anus, rare tumor, radiochemotherapy, quality of life

## Abstract

**Purpose:**

To describe the survival and toxicity outcome from a single-centre experience in patients with squamous cell cancer of the anal canal (SCC-AC), related to the impact of technological advances in diagnostics and radiation techniques.

**Material and Methods:**

A retrospective cohort study was performed after the approval of the institutional ethical committee (EK 478-21). We identified 142 patients in our registry, who received radical treatment for SCC-AC between 2000 and 2020. Fifty-five patients had FDG PET/CT for initial staging and target volume delineation, 87.33% received concomitant chemoradiotherapy (CRT), 64 patients were treated with 3-dimensional conformal radiotherapy (3DRT) between 2000-2009, and 78 patients with intensity-modulated radiotherapy (IMRT) between 2009-2020. Endpoints for the analysis included locoregional relapse-free survival (LRFS), disease-free survival (DFS), overall survival (OS), and cancer-specific survival (CSS). Acute and late toxicities were also reported.

**Results:**

At a median follow-up of 31.2 months, the median overall survival was 135 months, 5-year LRFS was 73.1%, 5-year DFS was 65.3%, and 5-year CSS was 75.3%. The use of IMRT was associated with shorter treatment duration. In the univariate analysis, IMRT was associated with significantly improved DFS and CSS for the whole cohort and significantly improved DFS, OS, and CSS for patients who received CRT. In the multivariate analysis, IMRT was associated with the improvement of all survival paraments. The use of FDG PET/CT did not translate into an improvement in the survival outcomes in both univariate and multivariate analyses. Grade-3 and more dermatological toxicities occurred less frequently, but hematological toxicities were more frequent in the IMRT-group. Late side effects and colostomies were less frequently reported in the IMRT group.

**Conclusion:**

The use of IMRT in the management of SCC-AC was associated with improvement of the oncological outcomes with improved toxicity profiles in this long-term single-centre experience.

## Introduction

Anal cancer is a rare disease with 2447 new cases diagnosed in 2018 in Germany, accounting for 0.4% of all cancer diagnoses. With 576 deaths, anal cancer contributes with 0.23% of cancer-related mortality for the same year (1).

In the era of the Human Papilloma Virus (HPV) epidemic, the incidence of anal cancer, as well as other HPV-related cancers, is steadily increasing in western countries. Fisch et al. found a positive HPV infection in 90% of women and 63% of men with anal cancer (2). The most common variant associated with anal cancer is HPV 16 (87%). Other variants include HPV 18 (7%) and HPV 33 (6%). Other risk factors include HIV infection, the history of anal intercourse, and organ transplant recipients (3).

Radiotherapy with concomitant chemotherapy (CRT) with fluoropyrimidines (5-FU) and Mitomycin c (MMC) is the primary treatment modality for patients with invasive anal cancer. This paradigm has not changed markedly since 1974 when Nigro et al. reported a complete pathological response (pCR) of 71% in their series of patients who were operated on after radiation with 30 Gy in 15 fractions concomitant with 5-FU and MMC, a similar tumor control rate of 80% was observed in patients with or without additional abdomino-perineal resection (APR) (4). The replacement of MMC with Cisplatin to spare hematological toxicities has been evaluated in two trials. However, Cisplatin worsened oncological outcomes in the RTOG 9811 trial. In the ACT II trial, Cisplatin was equally effective but increased toxicity (5, 6).

The feasibility of intensity-modulated radiotherapy (IMRT) in anal cancer management was evaluated in a single-arm phase II prospective study (RTOG 0529) and a favorable toxicity outcome compared to the historical standard of care (RTOG9811) could be demonstrated (7). Results from large retrospective cohorts using IMRT yielded favorable oncological outcomes compared to historical data (8–10). However, the comparison between IMRT and conformal 3-dimensional radiotherapy (3DRT) for oncological outcomes is not prospectively well evaluated.

Finally, positron-emission tomography (PET) has been routinely adopted more frequently in the staging and treatment planning of the disease. A recent meta-analysis showed high overall sensitivity and specificity of PET/CT in anal cancer diagnosis, 93% and 76%, respectively. Moreover, PET/CT resulted in upstage or downstage in 37.5% and 26.7%, respectively, which led to the modification of treatment plans in up to 59% (11).

The purpose of this study is to report from a long-term single-centre experience in the management of anal cancer, emphasizing the impact of the utilization of new technologies such as IMRT and PET/CT on the long-term survival and toxicities.

## Material and Methods

The institutional medical records were reviewed retrospectively for patients with anal cancer who received definitive radiotherapy or chemoradiation using 3DRT or IMRT between January 2000 and January 2021. Inclusion criteria for the analysis were: 1- histologically proven squamous cell cancer of anal canal (SCC-AC) 2- Stage I-III (AJCC 8^th^ edition). Exclusion criteria: 1- non-squamous cell cancer histology 2- Evidence of non-regional lymph node metastasis or distant metastasis at staging or treatment planning (Stage IV) 3- Radiation was applied as local palliative treatment. The retrospective analysis was approved by the institutional ethical committee (EK 478-21).

### Pretreatment Evaluation

All patients were diagnosed and evaluated through a multidisciplinary tumor board. Pretreatment evaluation included the patient’s history, a complete clinical examination, computer tomography (CT) of the chest and abdomen, and a proctosigmoidoscopy. [^18^F]2-fluoro-2-deoxy-d-glucose positron emission tomography-computed tomography ([^18^F]FDG PET/CT) and/or Magnetic Resonance Imaging (MRI) of the pelvis were carried out in a subset of patients. A gynecological examination was done in female patients to exclude other malignancies and/or tumor infiltration.

### Radiotherapy

A contrast-enhanced Planning CT was obtained in supine position with head and arm rest and knee fixation in 3 mm thickness. In the case of Planning PET/CT, both PET and CT scans were obtained in the radiation position in the same setting as previously described (12). Other investigations such as diagnostic CT or MRI of the pelvis were rigid registered in the planning system if available.

For CT-based target volume delineation, gross tumor volume (GTV) for the primary tumor was defined as the contrast-enhanced lesion in the anal and peri-anal region and for lymph node metastases as any pathologically enlarged lymph node with a short-axis diameter at least 10 mm. In the case of PET/CT-based target volume delineation, a volume of interest (VOI) first was defined around the lesion with high uptake in the anal canal, later the metabolic target volume for the primary tumor was automatically delineated to involve any volume in VOI with uptake at least 30% of the maximal standardized uptake value (SUVmax) (12), cases were revised in collaboration with an experienced nuclear medicine physician. The regional lymph node was considered positive if SUVmax was above 2.5. Elective lymph nodes included: inguinal, iliac, and mesorectal lymph nodes. The median dose to GTV was 55.8 Gy (50-56 Gy) based on T-stage, patient’s condition, and physician’s preference. The elective nodes received a dose of 41.4 or 45 Gy.

Patients were positioned in the supine position. A three-dimensional conformal treatment planning with 6-15 MV was standard in the years 2000-2009. A four-field or either a three-field (dorsolateral) or two-field (thunderbird, AP-PA) technique with additional electrons to the inguinal regions was used.

In 2009 intensity-modulated radiotherapy, initially, as step-and-shoot and later as volumetric modulated arc therapy (VMAT) was introduced as a standard treatment technique.

### Chemotherapy

The standard regimen for chemoradiation is 5-FU (1000 mg/m^2^) on days 1-4 and 29-32 and MMC 10 mg/m^2^ on days 1 and 29. Capecitabine with MMC was later approved as an alternative.

### Follow Up and Toxicity Documentation

Patients were examined at least once weekly during treatment and thereafter every three months for two years, followed by twice-yearly up to five years after radiation. Locoregional relapse-free survival (LRFS) was defined as the time span after CRT or RT until any disease persistence, progression, or recurrence in the pelvis or being censored. Disease-free survival (DFS) was defined as the time span till the occurrence of any type of relapse (locoregional or metastatic) or being censored. Overall survival (OS) was defined as the time span till death from any cause and cancer-specific survival (CSS) as the time span till death from anal cancer or treatment. Toxicities were documented by the treating physician and laboratory records from patients were saved in the local registry. For the study purpose, these were graded independently by 2 physicians (AM & MS) based on CTCAE Criteria 5.0. The discrepancies were separately evaluated by a 3rd physician (ME) for the final decision.

### Statistics

To detect differences in baseline characteristics and toxicities between subgroups, the Chi-Square test or the Mann-Whitney U test for non-parametric variables were applied. For univariate analysis, the Kaplan-Meier survival analysis with log-rank test was used to compare treatment subgroups and in addition a cox-regression analysis in multivariate analysis. The statistical analysis and graphs were done with the use of the R-software.

## Results

### Baseline Characteristics

We identified 159 patients with anal cancer who received radiotherapy in our registry. Sixty-five patients received PET/CT for staging and planning. Seventeen patients were excluded from the analysis due to: 1- upstaging to stage IV after PET/CT or staging CT (11 Patients) 2- non-squamous histology (6 patients: adenocarcinoma 3, sarcoma 2, and basal cell cancer 1).

A hundred and forty-two patients were eligible for the analysis, including 64 patients treated with 3DRT and 78 patients treated with IMRT. Median follow-up for all patients was 31.2 months (range 2.7-190 months) and for patients with 3DRT or IMRT 35.5 or 30.6 months, respectively (p-Value: 0.706). The comparison of baseline characteristics yielded a significantly increased number of regional lymph node metastases and, consecutively, a more advanced stage in patients with IMRT (**Table 1**). Only one patient in the 3DRT group and 54 patients in the IMRT group had a PET/CT-based target volume definition.

**Table 1:** Patient and baseline characteristics.

Characteristics	Conformal 3DRT n=64	IMRT n=78	*p*-Value
Sex			
Female Male	44 (69%) 20 (31%)	45 (58%) 33 (42%)	0.175^§^
Age	60	58.6	0.611^+^
cT-Status			
1 2 3 4	20 19 13 12	14 32 22 10	0.136^§^
cN-status			
0 1	53 11	41 37	0.0001^§^*
Stage (AJCC 8^th^, 2017)			
I IIA IIB IIIA IIIB IIIC	18 19 10 2 3 12	10 23 6 15 3 21	0.011^§^*
Grading			
G1 G2 G3 Undetermined	1 47 10 6	3 48 23 4	0.158^§^
P-16 status			
positive negative Undetermined	9 0 55	7 3 68	0.94^§^

For comparison of treatment groups, the Chi-Square^§^ and the Mann-Whitney U^+^ Tests were applied, * p-value < 0.05.

Concomitant chemotherapy was applied in 87.3% of patients, with the standard regimen (5-FU and MMC) in 93.5%, Capecitabine with MMC in 4%, and sole Fluoropyrimidine in 2.5%. Eighteen patients (12.7%) had sole radiotherapy, 7 patients with stage I based on the preference of the physician, and 11 patients with multiple comorbidities and contraindications for chemotherapy. The difference between the treatment groups was not statistically different (p-Value: 0.087) (**Table 2**).

**Table 2:** Treatment characteristics.

	Conformal 3DRT	IMRT	*p*-Value
PET-CT	1/64	54/78	<0.0001^§^*
CRT 5-FU-MMC Other	81.2% 78.1% 3.1%	92% 84.6% 7.7%	0.087^§^
RT-Duration (median)	46	41	<0.001^+^*
Treatment interruption > 2 days	25%	20.5%	0.331^§^
Dose Primary (Gy)	55	56	0.063^+^

For comparison of treatment groups, the chi-square^§^ and the Mann-Whitney U^+^ Tests were applied, *p-value < 0.05.

The median dose to the primary was 55 and 56 Gy in the 3DRT and IMRT groups, respectively. The median treatment time in the 3DRT group was significantly longer than in the IMRT group (46 versus 41 days, p <0.001). A non-significant trend of more treatment interruptions of more than 2 days was seen in the 3DRT group, (**Table 2**).

### Pattern of Failure

**Figure 1:**
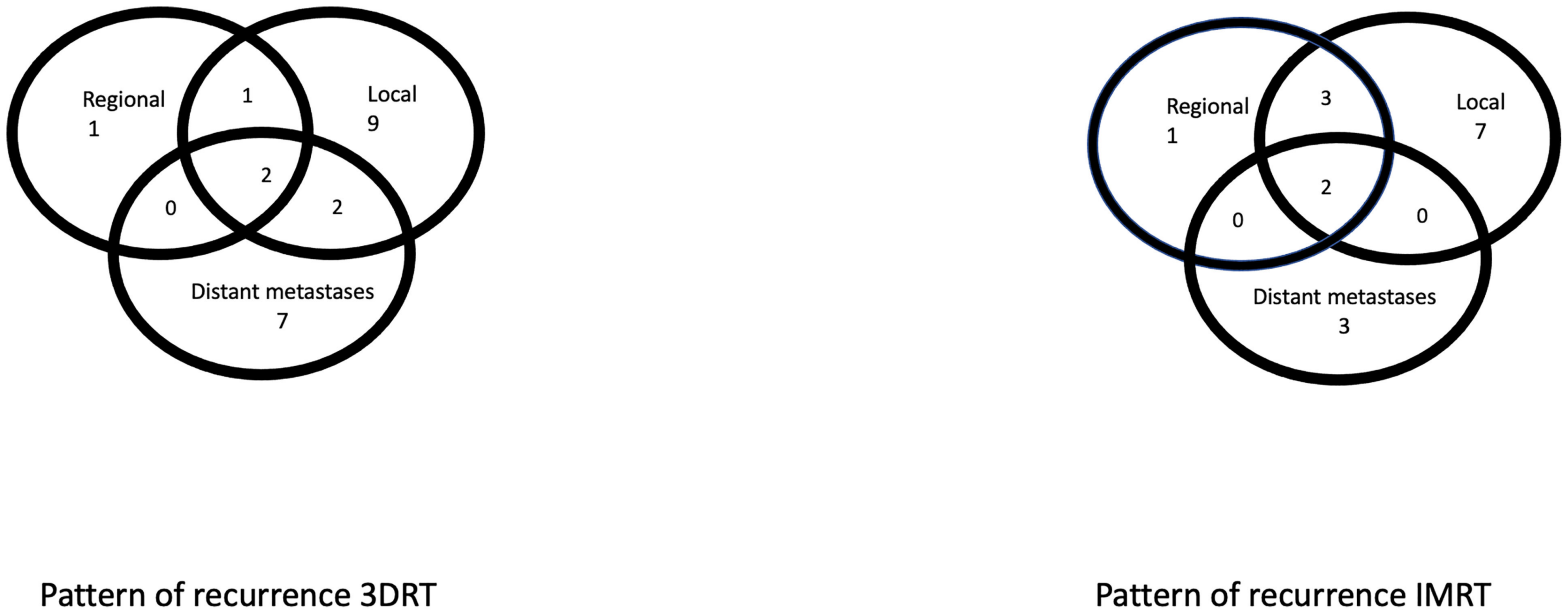
The pattern of failure in both 3DRT and IMRT groups.

The 3DRT group showed local, regional, and distant failure in 9 (14%), 1 (1.6%), and 7 (10.9%) patients and combined loco-regional, loco-distant, and loco-regional-distant in 1 (1.6%), 2 (3.1%) and 2 patients as the first manifestation of relapse, respectively. The IMRT group had a local, regional, and distant failure in 7 (9%), 1 (1.3%), and 3 (3.8%) patients, a combined loco-regional and loco-regional-distant in 3 (3.8%) and 2 (2.6%) patients, respectively (**Figure 1**).

### Survival Outcomes

The median overall survival for all patients was 135 months (95% CI: 112-157 months), the 5-year LRFS 73.1% (95% CI 65-83%), the 5-year DFS 65.3% (95% CI: 57-75%), the 5-year OS 74.7% (95% CI: 66-84%) and the 5-year CSS 75.3% (95% CI: 67-84%).

### Univariate Analysis

**Figure 2:**
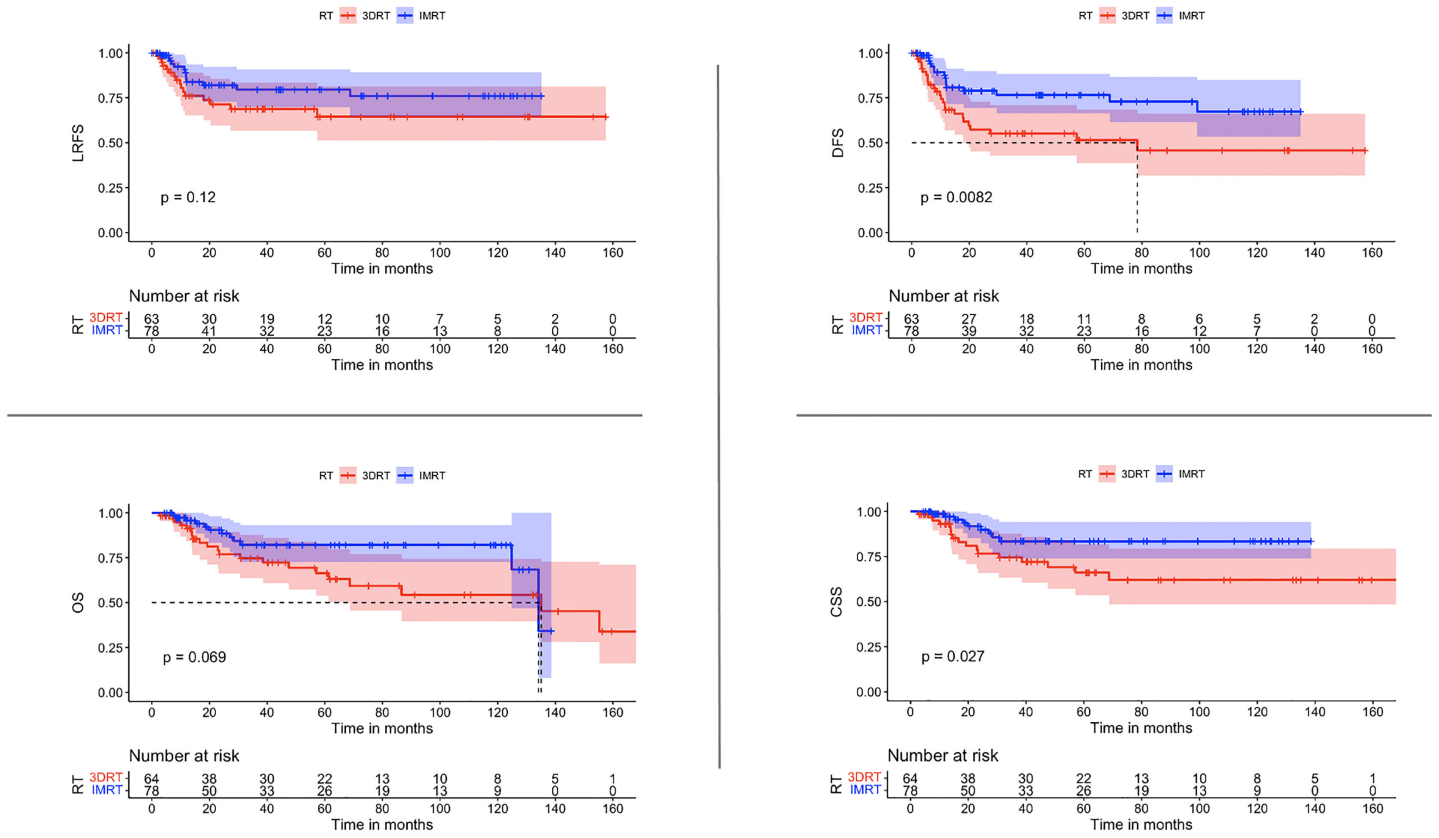
Comparisons of survival estimate of LRFS, DFS, OS, and CSS between both groups 3DRT and IMRT for the whole patients’ cohort represented by the Kaplan-Meier curves.

**Figure 3:**
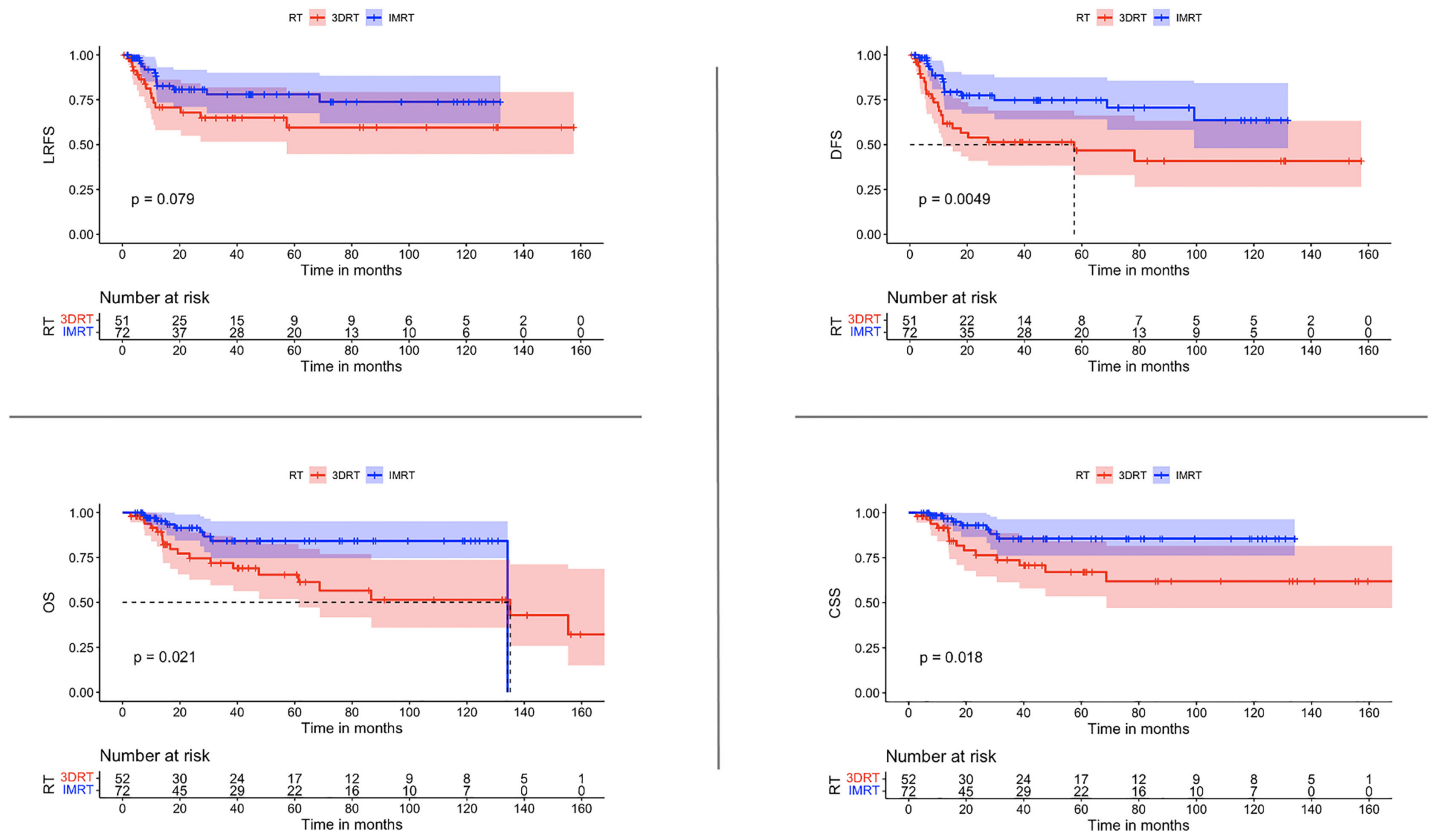
Comparisons of survival estimate of LRFS, DFS, OS, and CSS between both groups 3DRT and IMRT for the patients who received concomitant chemoradiation represented by the Kaplan-Meier curves.

**Figure 4:**
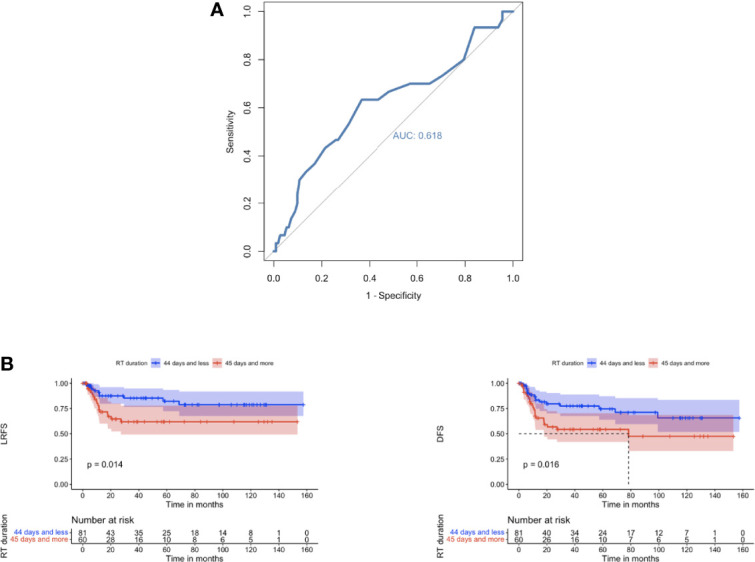
**(A)** ROC curve showing sensitivity and specificity for loco-regional recurrence after radiation/chemoradiation in relation to treatment duration. Area under the curve: 0.618. Ideal point: 44 days; sensitivity: 0.633 and specificity: 0.631. ROC, receiver-operating characteristic. **(B)** Comparison of LRFS and DFS for patients with treatment durations ≤ 44 days vs. ≥ 45 days represented by Kaplan–Meier curves.

The use of IMRT was associated with a significantly improved DFS (*p* 0.0082) and CSS (*p* 0.0027). After IMRT, LRFS and OS were not significantly improved (*p*-Value 0.12 & 0.069 respectively) (**Figure 2**). For those patients with concomitant chemotherapy, IMRT significantly improved DFS, OS, and CSS (*p*-Value: 0.049, 0.021, and 0.018, respectively) (**Figure 3**). Using a ROC-curve analysis between the treatment period and loco-regional recurrence, the AUC value was 0.615. A treatment time of more than 44 days, used as a cut point yielded a sensitivity and a specificity of 0.633 and 0.631, respectively, associated with the occurrence of loco-regional recurrences. In the univariate analysis, a treatment period ≥ 45 days showed a significantly higher locoregional recurrence risk and a decreased DFS (*p*-Value: 0.014 & 0.016, respectively) (**Figure 4**).

The use of a planning FDG PET/CT did not translate into a meaningful improvement in any of the survival parameters, OS, CSS, and LRFS: *p*-Value= 0.216, 0.2 & 0.53 respectively.

### Multivariate Analysis

Cox-regression analysis was applied for the multivariate analysis for all four survival parameters (**Table 3**). IMRT, younger age, early stages, and female sex resulted as independent variables for OS & CSS. IMRT was also an independent predictive factor for LRFS and DFS. PET/CT again was not a predictive factor for any of the survival parameters.

**Table 3:** Cox-regression analysis.

	LRFS	DFS	OS	CSS
Age	0.77	0.119	0.0002*	0.003*
Female sex	0.025*	0.461	< 0.0001*	< 0.0001*
Grade	0.663	0.277	0.894	0.229
Stage	0.278	0.003*	0.047*	0.047*
Dose to primary	0.561	0.117	0.380	0.748
PET-CT	0.520	0.059	0.474	0.216
IMRT	0.048*	< 0.0001*	0.011*	0.001*

*p-value < 0.05.

### Morbidity and Toxicity

Clinically relevant hematological acute toxicities (≥ Grade-3) were significantly higher in the IMRT group (p-value: 0.002). At the same time, relevant skin toxicity (≥ Grade-3) was reported to be significantly less in the IMRT group (p-value: 0.009), and the relevant gastro-intestinal (GI) toxicity (≥ Grade-3) was non-significantly reduced in the IMRT subgroup (p-Value 0.078). In the same way, the reduced risk of late side effects, including anal stenosis and fecal incontinence, favored the use of IMRT (p-Value 0.002 & 0.018, respectively) (**Table 4**).

**Table 4:** Morbidity and toxicity.

Morbidity/toxicity (≥ Grade 3)	Conformal 3DRT	IMRT	p-Value
Hematological toxicity (≥ G3)	12.2%	23.5%	0.002*
Derma-toxicity (≥ G3)	28%	6.8%	0.009*
GI-Toxicity (≥ G3)	18.8%	9.3%	0.078
Anal stenosis	9.3%	0	0.002*
Fecal incontinence	12.5%	7.1%	0.018*
Colostomy	26.6%	13.2%	0.035*

*p-value < 0.05.

IMRT is associated with a significantly lower colostomy rate for any reason (abdominoperineal resection, or disease/treatment-related complications) (p-value 0.035) (**Table 4**).

## Discussion

Even though only the feasibility and toxicity of IMRT in the treatment of SCC-AC were tested prospectively (7), IMRT has been widely implemented in clinical routine and replaced the 3DRT without prospective direct comparison for the oncological outcomes. In a pattern of care report for anal cancer patients from the National Cancer Database (NCDB), Hague et al. (13) demonstrated that by the year 2015, almost 96% of patients had been treated using IMRT. Similar results have been reported in a survey in German-speaking countries, with 97% of the centers reporting the use of IMRT in the management of SCC-AC. In addition, almost 12% reported the use of PET-CT as a routine examination in the treatment planning procedure (14). The possible reasons for the wide adoption of the IMRT are the potential to reduce the radiation dose to the organ at risk (OAR), possible shortening of treatment duration, and widespread availability of the technology, which can be easily implemented in practice. While several retrospective studies consistently reported the superiority of IMRT for toxicity outcomes (15, 16), only a few studies discussed the survival difference between the 2 modalities, and the results were conflicting (**Table 5**).

**Table 5:** Comparative survival outcomes between 3DRT and IMRT in SCC-AC.

Author/year of publication	Type of study	Number of patients	Survival outcomes
Bryant et al. (17)	retrospective database Study (VA database)	Total Number: 779 (Conventional RT: 403 & IMRT: 376)	No effect on the survival (p 0.18) and hematological toxicities (p 0.79)
Elson et al. (18)	retrospective database Study (NCBD database)	Total Number: 6814 (3DRT: 57.4% & IMRT: 42.6%)	IMRT associated with less treatment time and better 5-year OS (p 0.0036)
Lee et al. (19)	retrospective database Study (NCBD database)	Total Number: 6966 (3DRT: 55.5% & IMRT: 44.5%)	IMRT reduced the treatment time but no effect on 5-years OS (p 0.315)
Chuong et al. (20)	Single-centre retrospective study	Total Number: 89 (3DRT: 37, IMRT: 52)	No difference in the survival outcomes (OS, PFS, LRC: p> 0.1)
Koerber et al. (21)	Single-centre retrospective study	Total Number: 105 (3DRT 37, IMRT 68)	No difference in the survival outcomes (OS, PFS, LC: P> 0.05)
Possiel et al., et al. (22)	Single-centre retrospective study	Total Number: 149 (3DRT 87, IMRT 62)	IMRT Improved the CSS (p 0.018) & DC (p 0.012)

VA, Veterans Affairs; NCBD, National Cancer Database; 3DRT, conformal 3-dimensional radiotherapy; IMRT, intensity-modulated radiotherapy; OS, Overall survival; PFS, Progression-free survival; LRC, locoregional control; CSS, cancer-specific survival; DC, distant metastasis.

Such conflicting results reflect themselves on the reason for IMRT-recommendation in guidelines. NCCN and ESMO guidelines recommend IMRT in the management of SCC-AC solely due to the better toxicity profile (23, 24). On the other hand, the German guideline Program in Oncology (S3) recommends IMRT over 3DRT due to better toxicity outcomes, OS and CSS, however, they didn’t find a statistical significance for IMRT over 3DRT in LRFS or DFS (25).

In this retrospective analysis, we report a single-centre experience over 20 years of treating SCC-AC in a standardized manner. For the whole patients’ cohort, the median overall survival was 135 months (95% CI: 112-157 months), and the 5-years LRFS and DFS were 73.1% & 65.3%, respectively. The primary radiation technique from 2000 to 2009 was 3DRT, and after 2009, IMRT was utilized as the standard radiation modality. In addition, PET/CT has been implemented as a routine examination for staging and target volume definition.

In our series, the IMRT and 3DRT groups were balanced in most characteristics. However, in the IMRT group more regional lymph node metastases were seen, primarily due to the advances in diagnostics and the application of PET/CT in staging and planning. Considering the prognostic role of HPV status, unfortunately, we could not retrieve the HPV/p-16 status for the whole cohort as it was not routinely evaluated previously due to the lack of any therapeutic implications.

With the same median follow-up for the two groups, we observed an improvement in the DFS and CSS in IMRT patients and a trend for a decreased locoregional relapse rate with improved OS. Considering the crucial role of chemotherapy, we repeated the analysis only for the patients with concomitant chemotherapy. DFS, OS, and CSS were nevertheless significantly improved after IMRT, with a trend for improved LRFS. In the pattern of recurrence, we noticed an increased distant and slightly increased local failure rates in the 3DRT group. Interestingly, isolated regional failure has been diagnosed only once in each group.

The treatment duration in the 3DRT group was significantly longer and was associated with frequent treatment interruptions compared to IMRT. Such interruptions implied a longer duration of treatment, which is associated with a higher risk for locoregional relapse and worse disease-free survival if the treatment duration was more than 44 days.

The use of PET/CT did not show any relevant difference in the survival outcomes. However, this could be attributed to the relatively low number of patients who had PET/CT in the analysis.

In the multivariate analysis, three factors were explicitly associated with improved outcomes: female sex, early stage, and the use of IMRT. The phenomenon of better survival outcomes in female patients with SCC-AC has been previously reported (25). Reasons could be less aggressive tumor biology or a higher prevalence of HPV-related SCC-AC (2, 26). Nonetheless, we can assume the better oncological outcome after IMRT seems to be related to shorter treatment periods, fewer interruptions, and better target coverage.

In line with other studies (7, 27), we could prove that IMRT improved clinically relevant non-hematologic toxicities and late toxicities. However, our data showed an increase in hematologic toxicity (≥ G3) associated with the use of IMRT. A relevant cause could be the missing contouring of the pelvic bone marrow as OAR. Consequently, a larger but undefined volume of bone marrow was involved within the increased spatial dose bath with IMRT.

Of course, we identify several limitations of the current study that need to be openly addressed. Even when a prospective comparison for the oncological outcomes between the 2 modalities is unlikely, the retrospective nature of the study considers the main limitation. The imbalance in the lymph node involvement between both groups, although it was in favor of the 3DRT-group. Lastly, the undetermined HPV status for a major part of the cohort may hide an imbalance between the two groups which could affect the results greatly.

## Conclusion

The implementation of IMRT in the management of SCC-AC is associated with a reduction of treatment time and treatment compliance based on fewer toxicities. Altogether, an improvement in the oncological outcome for patients suffering from SCC-AC could be demonstrated with IMRT as an independent parameter in multivariate analysis.

## Data Availability Statement

The raw data supporting the conclusions of this article will be made available by the authors, without undue reservation.

## Ethics Statement 

The studies involving human participants were reviewed and approved by Faculty of Medicine, RWTH Aachen University. Written informed consent for participation was not required for this study in accordance with the national legislation and the institutional requirements.

## Author Contributions

Conception and design of the study (AM and ME), Data collection (AM, MS, and SK), Revision of data collection (ME and AH), Manuscript drafting and editing (AM, ME, and AH) critical revision of Manuscript (MS and SK). All authors contributed to the article and approved the submitted version.

## Conflict of Interest

The authors declare that the research was conducted in the absence of any commercial or financial relationships that could be construed as a potential conflict of interest.

## Publisher’s Note

All claims expressed in this article are solely those of the authors and do not necessarily represent those of their affiliated organizations, or those of the publisher, the editors and the reviewers. Any product that may be evaluated in this article, or claim that may be made by its manufacturer, is not guaranteed or endorsed by the publisher.

## Abbreviations

3DRT, three-dimensional radiation therapy; IMRT, intensity-modulated radiotherapy; 5-Fu, 5 Fluorouracil; MMC, Mitomycin C; AP-PA, anterior posterior-posterior anterior; VMAT, volumetric modulated arc therapy.
